# Impact of T_h_1 CD4 Follicular Helper T Cell Skewing on Antibody Responses to an HIV-1 Vaccine in Rhesus Macaques

**DOI:** 10.1128/JVI.01737-19

**Published:** 2020-02-28

**Authors:** Anil Verma, Brian A. Schmidt, Sonny R. Elizaldi, Nancy K. Nguyen, Korey A. Walter, Zoltan Beck, Hung V. Trinh, Ashok R. Dinasarapu, Yashavanth Shaan Lakshmanappa, Niharika N. Rane, Gary R. Matyas, Mangala Rao, Xiaoying Shen, Georgia D. Tomaras, Celia C. LaBranche, Keith A. Reimann, David H. Foehl, Johannes S. Gach, Donald N. Forthal, Pamela A. Kozlowski, Rama R. Amara, Smita S. Iyer

**Affiliations:** a The Center for Immunology and Infectious Diseases, UC Davis, Davis, California, USA; b Graduate Group in Immunology, UC Davis, Davis, California, USA; c Department of Microbiology, Immunology, and Parasitology, Louisiana State University Health Sciences Center, New Orleans, Louisiana, USA; d Henry M. Jackson Foundation for the Advancement of Military Medicine, Bethesda, Maryland, USA; e U.S. Military HIV Research Program, Laboratory of Adjuvant and Antigen Research, Walter Reed Army Institute of Research, Silver Spring, Maryland, USA; f Emory Department of Human Genetics, Emory University, Atlanta, Georgia, USA; g Duke Human Vaccine Institute, Duke University Medical Center, Durham, North Carolina, USA; h Department of Surgery, Duke University Medical Center, Durham, North Carolina, USA; i Department of Medicine, Duke University Medical Center, Durham, North Carolina, USA; j Department of Molecular Genetics and Microbiology, Duke University Medical Center, Durham, North Carolina, USA; k Department of Immunology, Duke University Medical Center, Durham, North Carolina, USA; l Nonhuman Primate Reagent Resource, MassBiologics, University of Massachusetts Medical School, Boston, Massachusetts, USA; m Division of Infectious Diseases, Department of Medicine, University of California, Irvine, School of Medicine, UC Irvine, Irvine, California, USA; n Department of Molecular Biology and Biochemistry, University of California, Irvine, School of Medicine, UC Irvine, Irvine, California, USA; o Department of Microbiology and Immunology, Emory University, Atlanta, Georgia, USA; p Yerkes National Primate Research Center, Emory University, Atlanta, Georgia, USA; q California National Primate Research Center, School of Veterinary Medicine, UC Davis, Davis, California, USA; r Department of Pathology, Microbiology, and Immunology, School of Veterinary Medicine, UC Davis, Davis, California, USA; Ulm University Medical Center

**Keywords:** antibody, CD4, T_fh_, vaccine, adjuvant

## Abstract

Generating durable humoral immunity through vaccination depends upon effective interactions of follicular helper T (T_fh_) cells with germinal center (GC) B cells. T_h_1 polarization of T_fh_ cells is an important process shaping the success of T_fh_-GC B cell interactions by influencing costimulatory and cytokine-dependent T_fh_ help to B cells. However, the question remains as to whether adjuvant-dependent modulation of T_fh_ cells enhances HIV-1 vaccine-induced antienvelope (anti-Env) antibody responses. We investigated whether an HIV-1 vaccine platform designed to increase the number of T_h_1-polarized T_fh_ cells enhances the magnitude and quality of anti-Env antibodies. Utilizing a novel interferon-induced protein 10 (IP-10)-adjuvanted HIV-1 DNA prime followed by a monophosphoryl lipid A and QS-21 (MPLA+QS-21)-adjuvanted Env protein boost (D_IP-10_ P_ALFQ_) in macaques, we observed higher anti-Env serum IgG titers with greater cross-clade reactivity, specificity for V1V2, and effector functions than in macaques primed with DNA lacking IP-10 and boosted with MPLA-plus-alum-adjuvanted Env protein (DP_ALFA_) The D_IP-10_ P_ALFQ_ vaccine regimen elicited higher anti-Env IgG1 and lower IgG4 antibody levels in serum, showing for the first time that adjuvants can dramatically impact the IgG subclass profile in macaques. The D_IP-10_ P_ALFQ_ regimen also increased vaginal and rectal IgA antibodies to a greater extent. Within lymph nodes, we observed augmented GC B cell responses and the promotion of T_h_1 gene expression profiles in GC T_fh_ cells. The frequency of GC T_fh_ cells correlated with both the magnitude and avidity of anti-Env serum IgG. Together, these data suggest that adjuvant-induced stimulation of T_h_1-T_fh_ cells is an effective strategy for enhancing the magnitude and quality of anti-Env antibody responses.

**IMPORTANCE** The results of the RV144 trial demonstrated that vaccination could prevent HIV transmission in humans and that longevity of anti-Env antibodies may be key to this protection. Efforts to improve upon the prime-boost vaccine regimen used in RV144 have indicated that booster immunizations can increase serum anti-Env antibody titers but only transiently. Poor antibody durability hampers efforts to develop an effective HIV-1 vaccine. This study was designed to identify the specific elements involved in the immunological mechanism necessary to produce robust HIV-1-specific antibodies in rhesus macaques. By clearly defining immune-mediated pathways that improve the magnitude and functionality of the anti-HIV-1 antibody response, we will have the foundation necessary for the rational development of an HIV-1 vaccine.

## Abstract (precis)

The results of the RV144 trial demonstrated that vaccination could prevent HIV transmission in humans and that longevity of anti-Env antibodies may be key to this protection. Efforts to improve upon the prime-boost vaccine regimen used in RV144 have indicated that booster immunizations can increase serum anti-Env antibody titers but only transiently. Poor antibody durability hampers efforts to develop an effective HIV-1 vaccine. This study was designed to identify the specific elements involved in the immunological mechanism necessary to produce robust HIV-1-specific antibodies in rhesus macaques. By clearly defining immune-mediated pathways that improve the magnitude and functionality of the anti-HIV-1 antibody response, we will have the foundation necessary for the rational development of an HIV-1 vaccine.

## INTRODUCTION

CD4 T follicular helper cells (T_fh_) are a specialized subset of CD4 T cells that migrate to germinal centers (GCs) within secondary lymphoid organs and provide growth and differentiation signals to GC B cells within a few days of immunization (1–3). GCs are populated by antigen (Ag)-activated, rapidly proliferating B cell clones, which rely on cytokines and costimulatory signals from T_fh_ cells to undergo immunoglobulin affinity maturation, class switch recombination, and differentiation to memory B cells and plasma cells (4–6). The maturation of GC B cells to plasma cells and the resulting long-lived humoral immunity hinge on effective T_fh_ help.

T_fh_ cells are heterogeneous and, depending on inflammatory signals during T cell priming, differentiate into T_h_1-, T_h_2-, and T_h_17-type T_fh_ cells (7, 8). T_h_ polarization of a T_fh_ cell influences the cytokine profile and costimulatory molecule expression, and several recent studies demonstrate that within a single vaccine modality, the relative proportion of T_fh_1, -2, or -17 subsets induced following antigen stimulation can influence the duration and functional quality of the antibody response (9). In the setting of influenza and human immunodeficiency virus type 1 (HIV-1) vaccination, the frequencies of vaccine-induced T_h_1-polarized, CXCR3-expressing T_fh_ cells correlate with improved antibody titers and enhanced antibody function following immunization (10–12). These data led us to postulate that by stimulating the production of T_h_1-T_fh_ cells via a tailored vaccine platform, humoral immunity against HIV-1 can be optimized in both duration and quality.

The RV144 trial found that waning serum anti-HIV-1 envelope (Env) IgG titers following vaccination corresponded to a decrease in vaccine efficacy (13, 14). Therefore, there is a critical need to identify strategies that will augment vaccine-mediated humoral immunity for a successful HIV-1 vaccine. In RV144, the development of antigen-specific CD4 T cells expressing interleukin-4 (IL-4) and CD40L, both of which are important for effective T_fh_ help for B cells (15, 16), positively correlated with anti-HIV-1 Env antibody titers. Furthermore, an increase in the production of HIV-specific CD4 T cells expressing IL-21, a T_fh_ cytokine that regulates plasma cell differentiation, was also observed (17–19). These data underscore the importance of CD4 T_fh_ cells in HIV-1 vaccine-induced antibody responses and suggest that identifying and targeting the optimal T_fh_ subset may be an effective strategy to improve the magnitude and longevity of anti-HIV-1 Env-specific antibodies.

Based on evidence that T_h_1-polarized T_fh_ cells correlate with higher antibody responses, we set out to investigate empirically whether an HIV-1 vaccine platform designed to increase the number of T_h_1-polarized T_fh_ cells would enhance the functional quality and magnitude of HIV-1 anti-Env antibodies.

Utilizing a novel interferon-induced protein 10 (IP-10)-adjuvanted HIV-1 DNA prime followed by a monophosphoryl lipid A and QS-21 (MPLA+QS-21)-adjuvanted Env protein boost (D_IP-10_ P_ALFQ_) in macaques, we show increased HIV-1 anti-Env-specific binding antibody in serum and mucosal compartments compared to vaccination with DNA lacking IP-10 and an MPLA-plus-alum-adjuvanted Env protein boost (DP_ALFA_). The D_IP-10_ P_ALFQ_ vaccine regimen augmented GC B cell responses and promoted T_h_1 gene expression profiles in GC T_fh_ cells. The number of GC T_fh_ cells positively correlated with both the magnitude and avidity of anti-Env-specific antibody responses. We report for the first time that adjuvants dramatically impact the IgG antibody subclass profile in rhesus macaques. We made the striking observation that while both vaccine regimens induced IgG1 antibodies to gp120, the DP_ALFA_ regimen generated much greater IgG4 responses. Together, these data show that by stimulating the production of T_h_1-T_fh_ cells during the prime and boost using an adjuvanted vaccine, we can enhance the magnitude and function of the anti-HIV-1 Env antibody response.

## RESULTS

### Vaccination regimen.

**FIG 1:**
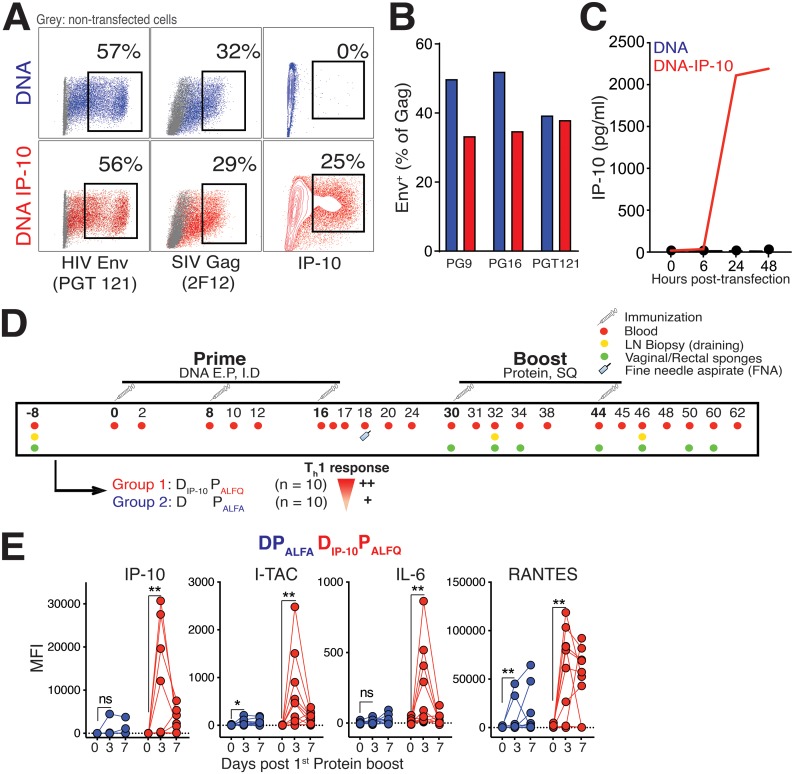
Immunization schedule for the subtype C HIV-1 envelope DNA (D) prime and protein boost (P) vaccine regimen. (A) Flow cytometric plots illustrating the expression of HIV Env, SIV Gag, and IP-10 by 293T cells transfected with DNA and DNA_IP-10_ plasmids. (B) Surface expression of HIV Env based on detection with a panel of monoclonal antibodies, as indicated. (C) IP-10 concentrations in supernatants of transfected 293T cells show the accumulation of IP-10 following transfection with DNA_IP-10_. (D) Immunization schedule. Two groups of 10 rhesus macaques each were immunized three times with DNA followed by two immunizations with protein. DNA was delivered intradermally, and 3 s later, electrical pulses were delivered around the injection site using the Ichor TriGrid array. Group 1 animals (*n* = 10) received the DNA plasmid expressing IP-10 and an ALFQ-adjuvanted C.ZA gp140 boost (D_IP-10_ P_ALFQ_). Group 2 (*n* = 10) animals were immunized with DNA and boosted with ALFA-adjuvanted C.ZA gp140 protein (DP_ALFA_). SQ, subcutaneous. (E) Induction of IP-10, I-TAC, IL-6, and RANTES in serum after the 1st protein immunization in both vaccine regimens. Significance was tested by a Mann-Whitney test (*, *P* < 0.05; **, *P* ≤ 0.01; ns, not significant).

Twenty female rhesus macaques were assigned to one of two experimental groups. For group 1 (*n* = 10), the D_IP-10_ P_ALFQ_ vaccine group, the T_h_1 chemokine IP-10, a ligand for and an inducer of CXCR3, was used as a molecular adjuvant to a DNA vaccine (D_IP-10_) to prime T_h_1-type T_fh_ cells. Group 2 (*n* = 10) animals received the same DNA vaccine without the adjuvant (Fig. 1). The DNA plasmid expressed simian immunodeficiency virus SIVmac239 Gag, protease, reverse transcriptase, Tat, and Rev and HIV C.1086 Env, and the D_IP-10_ plasmid additionally expressed rhesus IP-10. The DNA was delivered intradermally (i.d.) with electroporation (EP) in both experimental groups.

Prior to immunizing animals, we evaluated plasmid constructs using 293T cells. Forty-eight hours following transfection, cells were harvested, and the expression of HIV proteins was assessed by flow cytometry using the monoclonal antibodies PG9, PG16, and PGT121 for surface Env; 2F12 for intracellular SIV Gag; and J034D6 for intracellular IP-10. As illustrated in the flow plots in Fig. 1A, both constructs expressed comparable levels of Env and Gag proteins as determined by staining with PGT121 and 2F12, respectively. Cellular and secreted IP-10 as determined by intracellular cytokine staining (ICS) (Fig. 1A) and enzyme-linked immunosorbent assays (ELISAs) (Fig. 1C), respectively, was specific to the DNA_IP-10_ construct. When expressed as a percentage of Gag-positive (Gag^+^) cells, the expression of trimeric Env as determined by binding of the monoclonal broadly neutralizing antibodies PG9 and PG16, which bind the V1V2 loop, and the V3-binding monoclonal antibody PGT121 showed comparable expression levels across the two vaccine constructs.

Following DNA immunization, we used clade C C.ZA 1197MB gp140 protein adjuvanted with Army liposome formulation (ALF) liposomes containing monophosphoryl lipid A (MPLA) and a detoxified saponin derivative, QS-21 (ALFQ) (20), to boost T_h_1-primed responses (D_IP-10_ P_ALFQ_) (Fig. 1D). Group 2 animals received an unadjuvanted i.d., EP-delivered DNA prime and protein adjuvanted with an aluminum-adsorbed ALF formulation (ALFA) (21), wherein the protein was adsorbed to aluminum hydroxide (AH) and then added to ALF (DP_ALFA_). Blood was collected at weeks −8 and 0 of vaccination and at weeks 1, 2, 4, 8, 18, and 20 following each vaccination, as indicated. Fine-needle aspirates of lymph nodes (LNs) or LN biopsy specimens (draining) were collected to examine GC responses, and rectal and vaginal secretions were sampled to assess mucosal antibodies.

To confirm that the D_IP-10_ P_ALFQ_ vaccine regimen induced relatively higher T_h_1-biased inflammatory responses, we evaluated the induction of CXCR3 ligands in serum using a flow-based Legendplex assay at days 0, 3, and 7 after the 1st protein boost. The data showed higher relative inductions of IP-10 and interferon-inducible T cell alpha chemoattractant (I-TAC) in the ALFQ-adjuvanted animals (*P* < 0.01) (Fig. 1E). Monokine induced by gamma, another CXCR3 ligand, was not induced following the 1st protein boost in either vaccine regimen (data not shown). We also observed a significant induction of IL-6 following the ALFQ protein boost. The induction of the chemokine regulated upon activation, normal T cell expressed, and secreted (RANTES) in both vaccine groups indicated the presence of activated CD4 and CD8 T cells following vaccination. In all, these data showed a higher relative magnitude of T_h_1 chemokines in the D_IP-10_ P_ALFQ_ vaccine regimen.

### The D_IP-10_ P_ALFQ_ vaccine induces robust and durable anti-Env antibody with cross-clade breadth.

**FIG 2:**
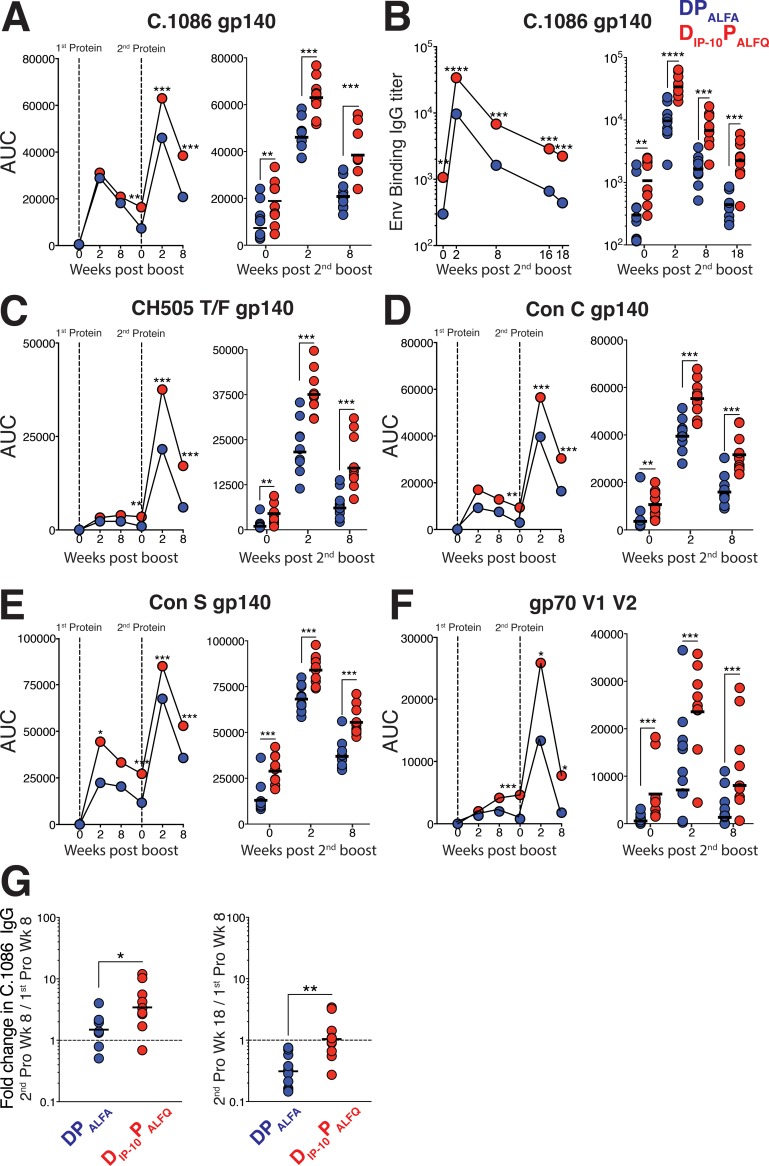
The D_IP-10_ P_ALFQ_ vaccine induces robust anti-Env serum IgG antibody with cross-clade breadth. (A) Kinetics of the C.1086 anti-gp140 IgG response measured by a BAMA in serum at weeks 0, 2, and 8 following each protein boost. The right panel shows scatter plot values for each animal at weeks 0, 2, and 8 after the 2nd protein boost. (B) Kinetics of the C.1086 gp140-specific anti-Env IgG response measured by an ELISA after the 2nd protein boost. The right panel shows titers for each individual animal. (C to F) A BAMA was used to measure responses against CH505 gp140 (C), Con C gp140 (D), Con S gp140 (E), and gp70-V1V2 Case A2 (F). (G) Fold changes in antibody titers at the indicated time points after the 2nd protein boost relative to the 1st. Kinetic data show geometric means. Vertical dotted lines show immunization time points. In dot plots, geometric means are indicated as horizontal lines. Statistical significance across vaccine regimens was tested using the Mann-Whitney U test (*, *P* ≤ 0.05; **, *P* ≤ 0.01; ***, *P* ≤ 0.001; ****, *P* ≤ 0.0001).

To ascertain whether the induction of greater-magnitude T_h_1 inflammatory responses elicited anti-Env antibody responses of different magnitudes between the vaccine regimens, we first evaluated responses against C.1086 gp140 Env using a binding antibody multiplex assay (BAMA) (22). We have previously shown that the transient extrafollicular plasmablast response contributes to peak serum IgG antibody titers following the boost, while titers at week 8 and beyond are mainly plasma cell derived (12). Therefore, we assessed antibody levels at weeks 0, 2, and 8 following each of the protein boosts to capture both extrafollicular (week 2) and plasma cell-derived (week 8 and beyond) titers. The data showed robust induction of anti-C.1086 Env responses following the 1st protein immunization in all 20 animals and potent recall of memory B cells following the 2nd protein immunization as evidenced by a robust boost in antibody responses (Fig. 2A). Strikingly, Env ALFQ-boosted animals developed significantly higher responses against C.1086 gp140; the median area under the concentration-time curve (AUC) values in the ALFA and ALFQ vaccine groups were 7,496 and 20,301 at week 0 (*P* < 0.01), 46,481 and 63,469 at week 2 (*P* < 0.001), and 20,714 and 36,709 at week 8 (*P* < 0.0001) after the 2nd protein boost, respectively.

We confirmed these findings by using an independent ELISA to explore C.1086 gp140 anti-Env antibody kinetics after the 2nd protein boost (Fig. 2B). The assay revealed that anti-Env titers exhibited a median 5-fold increase at week 2 after the 2nd protein immunization relative to week 0, indicating a successful booster response. In affirmation of the BAMA data, antibody titers were significantly higher in the D_IP-10_ P_ALFQ_ group than in the DP_ALFA_ group at all time points after the 2nd protein boost.

We next assessed the breadth of the serum IgG antibody response and found that AUC values against CH505 subtype C Env were also significantly higher in the D_IP-10_ P_ALFQ_ group than in the DP_ALFA_ group (*P* < 0.01) (Fig. 2C). Similarly, increased responses against the Con S (group M consensus) and Con C gp140 proteins at week 2 following the 2nd protein boost in the D_IP-10_ P_ALFQ_ group were sustained at week 8, demonstrating a greater induction of antibodies with cross-clade breadth using the D_IP-10_ P_ALFQ_ vaccine regimen (*P* < 0.05) (Fig. 2D and E). We also assessed binding to gp120 V1V2 loops from isolate Case A2, scaffolded on murine leukemia virus (MLV) gp70, at weeks 2 and 8 and found that a significantly higher specificity for these important regions was induced by the D_IP-10_ P_ALFQ_ vaccine regimen following the second protein boost (*P* < 0.05) (Fig. 2F).

Based on the significantly elevated anti-Env antibody responses in the D_IP-10_ P_ALFQ_ vaccine regimen, we sought to quantify the decline in antibody magnitude. To this end, we calculated fold changes in titers at weeks 8 and 18 following the 2nd protein boost relative to titers at week 8 after the 1st protein boost. The significantly higher titers at week 8 (mean of 1.7-fold in the DP_ALFA_ group versus 4.5-fold in the D_IP-10_ P_ALFQ_ group; *P* < 0.05) and week 18 (mean of 0.3-fold in the DP_ALFA_ group versus 1.3-fold in the D_IP-10_ P_ALFQ_ group; *P* < 0.01) after the 2nd protein boost in D_IP-10_ P_ALFQ_-vaccinated animals suggested that the D_IP-10_ P_ALFQ_ vaccine regimen was effective at enhancing the magnitude of anti-HIV-1 Env serum IgG titers (Fig. 2G). Together, these data show that the D_IP-10_ P_ALFQ_ group had higher induction of cross-clade breadth and elicited strong binding to a gp70-V1V2 protein and enhanced antibody responses relative to the DP_ALFA_ group.

### The D_IP-10_ P_ALFQ_ vaccine elicits high-avidity anti-Env antibody with ADCC and ADP activities.

**FIG 3:**
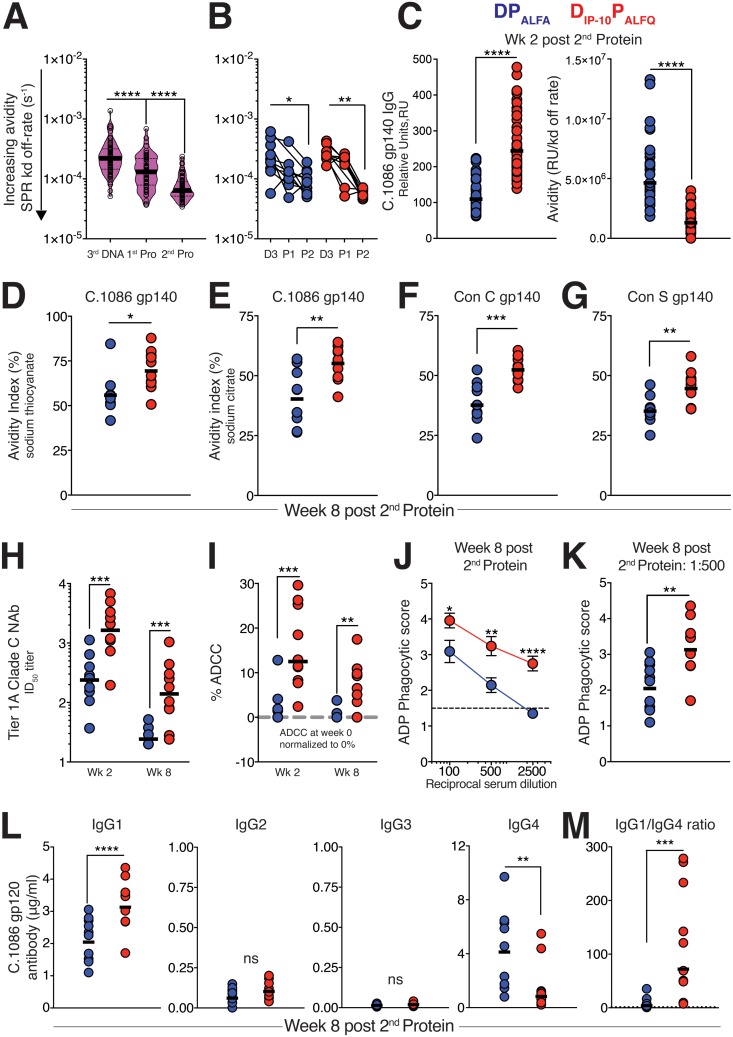
The D_IP-10_ P_ALFQ_ vaccine elicits high-avidity anti-Env antibody with ADCC and ADP activities. (A) Surface plasmon resonance (SPR) was used to determine the avidity index (AI) in serum 2 weeks after the third DNA immunization and each protein boost using C.1086 gp140 protein immobilized onto sensor chips. Violin plots show medians (bold lines) and interquartile ranges (dashed lines) in both vaccine groups, with each sample run in quadruplicate. Lower values indicate higher avidities. (B) SPR-based AI values for the two vaccine regimens over time. (C) Significantly higher IgG values for D_IP-10_ P_ALFQ_ 2 weeks after the 2nd protein boost (expressed as relative units, as measured by SPR) and higher avidity after normalizing avidity to gp140 IgG RU. Lower values indicate higher avidities. (D and E) AI measured against C.1086 gp140 using 2 M sodium thiocyanate (D) and 0.1 M sodium citrate (E) at week 8 after the 2nd protein boost. (F and G) AIs against Con C (F) and Con S (G) gp140 measured using 0.1 M sodium citrate at week 8 after the 2nd protein boost. (H) Serum neutralizing antibodies (NAb) were assessed against tier 1A (MW965.26) pseudovirus, and the 50% infective dose (ID_50_) was determined. (I) ADCC activity against SHIV CH505-infected target cells. Data are presented with week 0 serum ADCC values normalized to 0% (dashed gray line). (J) ADP using clade C Du151 gp120-coated beads was measured using sera from week 8 after the 2nd protein boost at serum dilutions ranging from 1:100 to 1:2,500. (K) Individual ADP scores at a 1:500 serum dilution. (L) C.1086 gp120-specific IgG subclass analysis was performed by an ELISA using serum collected 8 weeks after the 2nd protein boost. (M) IgG1/IgG4 ratios across vaccine groups at week 8 after the 2nd protein boost. Statistical significance across vaccine regimens was examined using the Mann-Whitney U test, and within-group differences over time were tested using a Wilcoxon matched-pairs signed-rank test (*, *P* ≤ 0.05; **, *P* ≤ 0.01; ***, *P* ≤ 0.001; ****, *P* ≤ 0.0001).

Next, we quantified the avidity of IgG binding antibodies (as a disassociation constant [*K_d_*]) in sera collected 2 weeks after the final DNA prime and after each of the protein boosts using surface plasmon resonance (SPR) for C.1086 gp140 protein (23). The data showed that gp140-specific antibodies reached higher avidity with each sequential immunization in both vaccine groups (*P* < 0.0001) (Fig. 3A and B). Consistent with the ELISA results (Fig. 2), SPR-based IgG measurements, expressed as relative units (RU), showed significantly higher gp140 IgG levels in the D_IP-10_ P_ALFQ_ vaccine group (*P* < 0.0001) (Fig. 3C). Therefore, we normalized avidity measurements to gp140 binding measurements and observed increased avidities in the D_IP-10_ P_ALFQ_ vaccine group relative to the DP_ALFA_ group, which was suggestive of a more productive GC reaction in the D_IP-10_ P_ALFQ_ vaccine group (*P* < 0.0001) (Fig. 3C). To confirm that higher-avidity antibodies in the D_IP-10_ P_ALFQ_ vaccine group were sustained, we determined avidity 8 weeks following the 2nd protein boost using a 2 M sodium thiocyanate displacement ELISA with C.1086C gp140 antigen (12). The data showed sustained induction of higher-avidity antibodies in the D_IP-10_ P_ALFQ_ group (*P* < 0.05) (Fig. 3D), which was further corroborated by a 0.1 M sodium citrate ELISA (*P* < 0.01) (Fig. 3E). Notably, higher-avidity antibodies against Con C and Con S gp140 proteins were also induced by the D_IP-10_ P_ALFQ_ vaccine regimen (*P* < 0.01) (Fig. 3F and G).

After establishing the induction of higher-avidity antibodies in the D_IP-10_ P_ALFQ_ vaccine group, we next evaluated the capacity of immune sera to neutralize HIV-1 using the classic TZM-bl assay (12). We detected robust activity against MW965.26, a subtype C tier 1A variant (Fig. 3H), whereas neutralization of tier 1B and tier 2 isolates was sporadic (data not shown) (24). The data showed higher induction of tier 1A neutralizing antibodies in the D_IP-10_ P_ALFQ_ vaccine group (50% infective dose [ID_50_] ranges at week 2 after the 2nd protein boost of 37 to 1,126 for the DP_ALFA_ group and 195 to 4,977 for the D_IP-10_ P_ALFQ_ group; *P* < 0.01). These titers dropped to an ID_50_ value of 20 in the DP_ALFA_ group but were maintained at values of between 24 and 1,057 in the D_IP-10_ P_ALFQ_ group (*P* < 0.001). To assess the generation of Fc-mediated antibody effector responses, we measured antibody-dependent cellular cytotoxicity (ADCC) and antibody-dependent phagocytosis (ADP) triggered by the engagement of the Fc receptors on antibody-bound target cells by innate cells (25–27). ADCC was assessed by measuring the killing of simian-human immunodeficiency virus clade C CH505 (SHIV.C.CH505)-infected CEM.NKR target cells by a rhesus CD16^+^ (FcγR3) NK cell line in the presence of immune serum. As shown in Fig. 3I, serum from D_IP-10_ P_ALFQ_-vaccinated animals demonstrated significantly greater ADCC activity at week 2 and week 8 after the 2nd protein boost than did serum from DP_ALFA_-immunized animals (*P* < 0.01). Serum collected from D_IP-10_ P_ALFQ_-vaccinated animals at week 8 after protein boost 2 also demonstrated significantly greater phagocytosis of C.1086 gp120-coated beads by the CD32^+^ (FcγR2) and CD64^+^ (FcγR1) THP-1 monocytic cell line (Fig. 3J and K). To determine if the adjuvants given to animals in the vaccine groups generated different rhesus IgG subclass antibody repertoires, we quantified C.1086 gp120-specific IgG1, IgG2, IgG3, and IgG4 by an ELISA. We found that the levels of IgG2 and IgG3 antibodies were extremely low and did not differ between groups (Fig. 3L). However, D_IP-10_ P_ALFQ_-vaccinated animals had higher levels of gp120-specific IgG1 (*P* < 0.0001) (Fig. 3L), while DP_ALFA_-vaccinated animals had higher levels of gp120-specific IgG4, resulting in a markedly elevated IgG1/IgG4 ratio in the D_IP-10_ P_ALFQ_ vaccine group (*P* < 0.001) (Fig. 3M). The IgG4 detection antibody (clone 78A) showed minimal cross-reactivity to IgG1 and IgG3 subclass antibodies, indicating the specificity of the antibody for rhesus IgG4 (data not shown). These results are consistent with reports that antibodies of the IgG1 subclass are the most abundant in rhesus macaques (28, 29).

### The D_IP-10_ P_ALFQ_ vaccine elicits robust anti-Env antibody in vaginal and rectal mucosal compartments.

**FIG 4:**
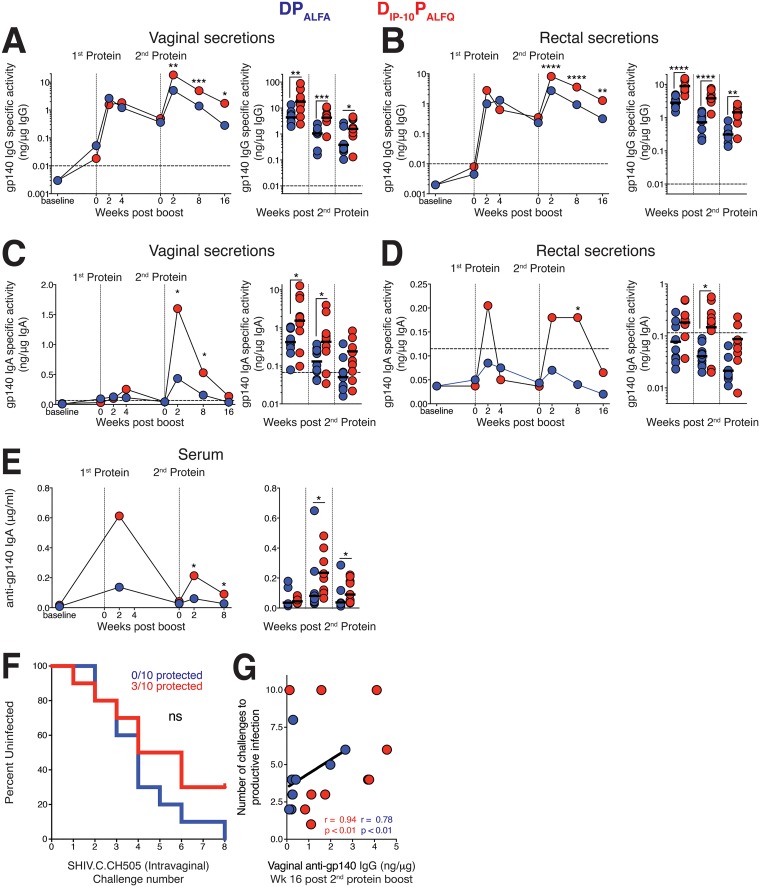
The D_IP-10_ P_ALFQ_ vaccine elicits robust anti-Env antibody in vaginal and rectal mucosal secretions. Concentrations of anti-C.1086 gp140 IgG and IgA in secretions were measured by a BAMA and adjusted in accordance with the total IgG and IgA, respectively, to obtain the specific activity. (A to D) Development of gp140-specific IgG (A and B) and IgA (C and D) in vaginal and rectal secretions. (E) Kinetics of the C.1086 gp140-specific IgA response in serum. Horizontal dashed lines represent the cutoff for significance. Kinetic data show geometric means. Vertical dotted lines show immunization time points. In dot plots, data from after the 2nd protein boost are shown, and geometric means are indicated as horizontal lines. (F) Kaplan-Meier plot showing acquisition rates following eight repeat intravaginal challenges with SHIV.C.CH505. (G) Vaginal anti-gp140 IgG concentrations at week 16 after the 2nd protein boost correlated with a delay in acquisition in infected animals in both vaccine regimens (D_IP-10_ P_ALFQ_ vaccine regimen, *n* = 7; DP_ALFA_ vaccine regimen, *n* = 10). Statistical significance was tested using an unpaired, two-tailed Mann-Whitney U test (*, *P* ≤ 0.05; **, *P* ≤ 0.01; ***, *P* ≤ 0.001; ****, *P* ≤ 0.0001), and correlations with a Spearman rank correlation.

Having established the induction of higher serum IgG antibody titers in D_IP-10_ P_ALFQ_-vaccinated animals, we next sought to determine whether mucosal anti-Env antibodies were also correspondingly increased. To this end, we assayed rectal and vaginal secretions for C.1086 gp140-specific IgG and IgA antibodies at baseline and longitudinally after each of the protein boosts. We next asked whether either vaccine regimen induced mucosal antibody responses; we focused on quantifying concentrations following the 1st protein boost, a time point when mucosal IgG and IgA concentrations are above baseline (background) levels in the majority of the animals. The appearance of gp140-specific IgG in secretions closely mimicked the kinetics of the serum IgG antibody response, with each protein boost increasing the levels of Env-specific IgG antibodies in vaginal and rectal secretions (Fig. 4A and B). As in serum, the D_IP-10_ P_ALFQ_ vaccine regimen generated higher levels of specific IgG in secretions than did the DP_ALFA_ vaccine. The gp140-specific IgA in vaginal and rectal secretions was also increased to a greater extent by the D_IP-10_ P_ALFQ_ vaccine regimen (Fig. 4C and D). Notably, at week 16 after the 2nd protein boost, vaginal IgA antibodies were still above the limit of detection in most D_IP-10_ P_ALFQ_-vaccinated animals but in only 2 of 10 DP_ALFA_-vaccinated animals. Analysis of gp140-specific IgA in serum revealed higher induction in the D_IP-10_ P_ALFQ_ group (Fig. 4E). However, the kinetics of the serum IgA response in D_IP-10_ P_ALFQ_- as well as DP_ALFA_-vaccinated animals differed strikingly from the mucosal IgA responses, especially in the reproductive tract (Fig. 4C and D), suggesting that a true mucosal (locally derived) IgA response was generated in these animals. This was most evident after the 2nd protein boost, when vaginal IgA antibodies to gp140 were found to be dramatically increased but serum IgA antibodies were reduced (Fig. 4A and C). Together, these data demonstrate that the D_IP-10_ P_ALFQ_ vaccine regimen was more effective than the DP_ALFA_ regimen for generating higher-magnitude Env binding antibodies in serum and secretions as well as serum IgG antibodies with greater breadth, avidity, and function.

Next, we determined whether the relatively higher concentrations of anti-gp140 antibody in mucosal secretions in the D_IP-10_ P_ALFQ_ vaccine group might result in delayed acquisition against the clade C transmitted/founder (T/F) virus SHIV.C.CH505. To this end, we challenged monkeys intravaginally with eight repeat, low-dose inoculations of SHIV.C.CH505 at week 20 after the 2nd protein boost. While we observed no significant differences in the delay in acquisition between the vaccine groups, 3 of 10 animals in the D_IP-10_ P_ALFQ_ vaccine group were protected, relative to 0 of 10 animals in the DP_ALFA_ group (Fig. 4F). We observed that the mucosal gp140 IgG antibody concentration at week 16 after the 2nd protein boost was a correlate of protection, with higher concentrations correlating with delayed acquisition in infected animals in each of the vaccine groups (*r* = 0.94 and *P* < 0.01 for the D_IP-10_ P_ALFQ_ vaccine group [*n* = 10]; *r* = 0.78 and *P* < 0.01 for the DP_ALFA_ vaccine group [*n* = 10]) (Fig. 4G).

### The D_IP-10_ P_ALFQ_ vaccine induces Env-specific T_fh_ cells and GC T_fh_ cells with distinctive T_h_1 signatures.

**FIG 5:**
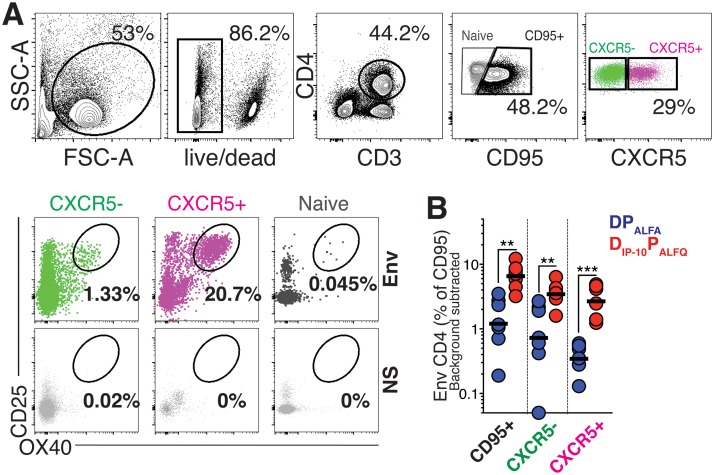
The D_IP-10_ P_ALFQ_ vaccine induces Env-specific T cells and T_fh_ cells in blood. (A) Gating strategy to identify CXCR5^+^ OX40^+^ CD25^+^ Env-specific T_fh_ cells within PBMCs after stimulation with both whole C.1086 gp140 protein and pooled overlapping peptides representing Con C gp140. The flow plot illustrates responses following stimulation with Env or volume-controlled DMSO (NS). SSC, side scatter; FSC, forward scatter. (B) Frequency of Env-specific CD4 T cells at week 1 after the 1st protein boost.

The D_IP-10_ P_ALFQ_ vaccine promoted anti-Env antibody magnitude and functionality following the 1st protein boost. Based on this finding, we wanted to determine whether this vaccine regimen also correspondingly enhanced T_fh_ cells in the periphery and LNs. To this end, we first assessed whether higher frequencies of Env-specific T_fh_ cells were induced in blood 7 days after the 1st protein boost, corresponding to the peak of the effector response. Peripheral blood mononuclear cells (PBMCs) were stimulated with overlapping peptide pools representing Con C gp140 together with HIV-1 C.1086 Env gp140 protein. The induction of the activation markers CD25 and OX40 was assessed by flow cytometry after stimulation (Fig. 5A, flow plot) (30). The analysis revealed a higher frequency of Env-specific CD4 T cells in the circulation of D_IP-10_ P_ALFQ_ animals. When expressed as a percentage of CD95^+^ CD4 T cells, median frequencies of Env-specific CD4 T cells were on average 10-fold higher in the D_IP-10_ P_ALFQ_ group, indicative of a higher-magnitude Env-specific T_fh_ response (*P* < 0.001) (Fig. 5B). In all, these data showed robust recall responses following the 1st protein boost, with a higher relative magnitude of Env-specific T_fh_ cells in the D_IP-10_ P_ALFQ_ vaccine regimen.

**FIG 6:**
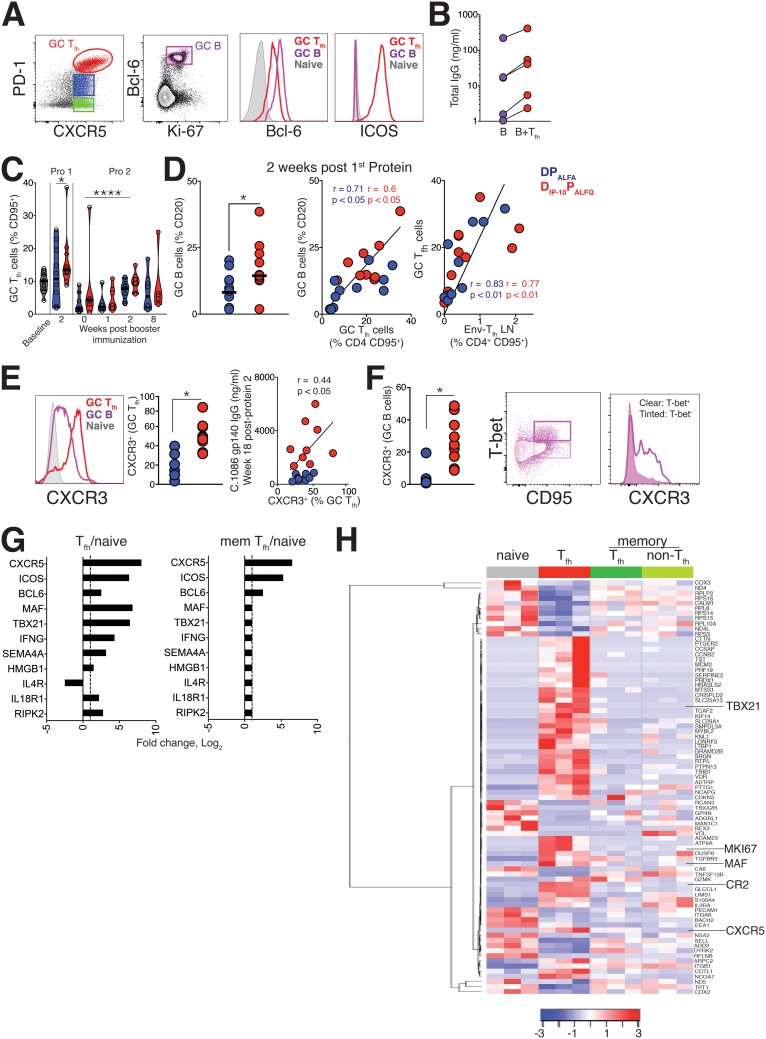
The D_IP-10_ P_ALFQ_ vaccine induces GC T_fh_ cells with distinctive T_h_1 signatures. (A) Gating strategy to identify GC T_fh_ cells and GC B cells in LNs 2 weeks after the 1st protein boost. Histograms show higher relative expression levels of Bcl-6 and ICOS in GC T_fh_ cells than in naive CD4 T cells. Expression in GC B cells is also shown. (B) Total IgG was measured in *ex vivo* coculture experiments with sorted GC T_fh_ cells and autologous LN B cells to demonstrate the B helper capacity of the T_fh_ cells. (C) Frequencies of GC T_fh_ cells in LNs at specified time points. Symbols indicate significant differences from the baseline for protein 1 and from day 0 for protein 2. (D) Dot plot illustrating higher frequencies of GC B cells in the T_h_1 vaccine group and correlations between frequencies of GC T_fh_ cells and GC B cells or Env-specific T_fh_ cells in LNs. Frequencies of Env-specific CD4 T cells in LNs (right) correlate with GC T_fh_ cells. (E, left) Histogram illustrating relative CXCR3 expression in GC T_fh_ cells and GC B cells. (Right) Dot plots showing significantly higher CXCR3 expression levels on GC T_fh_ cells in the T_h_1 vaccine group. Serum antibody titers at week 18 after the 2nd protein boost correlate with the frequency of GC T_fh_ cells and the proportion of CXCR3-expressing GC T_fh_ cells 2 weeks after the 1st protein boost. (F, left) Dot plot showing significantly higher CXCR3 expression levels on GC B cells from animals in the T_h_1 vaccine group. (Right) Flow plots illustrating higher expression levels of CXCR3 on T-bet^+^ memory B cells. (G) Log fold change values for key T_fh_ and T_h_1 genes in T_fh_ and memory (mem) T_fh_ cells in lymph nodes of T_h_1-vaccinated animals (adjusted *P* value of <0.05). (H) Heat map showing the expression of genes differentially expressed in T_fh_ cells relative to naive cells across four sorted CD4 subsets. Blue and red represent high and low relative log_2_ gene expression values, respectively. For the construction of heat maps, log_2_ gene expression values (counts per million) for the most differentially expressed genes in the comparison of T_fh_ versus naive cells were selected by the threshold of an adjusted *P* value of ≤0.05. Statistical significance was tested using an unpaired, two-tailed Mann-Whitney U test. Spearman coefficient-of-correlation values were computed to determine associations (*, *P* < 0.05; ****, *P* ≤ 0.0001).

Next, we assessed LN responses using biopsy specimens collected on day 14 after the 1st protein boost and identified GC T_fh_ cells as CXCR5^+^ PD-1^+++^ cells (Fig. 6A, red population) and GC B cells as Ki-67^+^ Bcl-6^+^ CD20 cells. As expected, GC T_fh_ cells expressed Bcl-6 and ICOS, and consistent with the functional ability of T_fh_ cells (12), our *ex vivo* analysis of sorted GC T_fh_ cells revealed their capacity to support IgG production by autologous LN B cells (Fig. 6B). Evaluation of GC T_fh_ frequencies over the course of immunization revealed a significant induction of GC T_fh_ cells 2 weeks after protein boost 1 relative to baseline and significantly higher frequencies 2 weeks after protein boost 2 than at week 0 of protein boost 2 (Fig. 6C).

While the frequencies of GC T_fh_ cells were not significantly different between experimental groups, we found that GC B cell frequencies were significantly higher in the D_IP-10_ P_ALFQ_ vaccine regimen (*n* = 10 animals in each group following the 1st protein boost; a median for the DP_ALFA_ group of 14.2% [of CD20^+^ cells] versus a median for the D_IP-10_ P_ALFQ_ group of 25% [*P* < 0.05]), and the frequency of GC T_fh_ cells strongly correlated with GC B cell responses (Fig. 6D). Importantly, Env-specific T_fh_ cell frequencies in the LNs directly correlated with GC T_fh_ cell frequencies but not memory T_fh_ cells, indicating that GC T_fh_ cells were enriched for vaccine-induced follicular cells (*P* < 0.0001) (Fig. 6D). Next, we assessed the expression of CXCR3, which is heterogeneously expressed by GC T_fh_ cells (Fig. 6E), and found higher expression levels of CXCR3 on GC T_fh_ cells in the D_IP-10_ P_ALFQ_ group. We observed that the frequency of CXCR3^+^ T_fh_ cells within the GC was directly associated with gp140 serum antibody titers at week 18 after the 2nd protein boost (*r* = 0.44; *P* < 0.05) (Fig. 6E). Examination of GC B cells showed elevated CXCR3 expression in GC B cells from the D_IP-10_ P_ALFQ_ vaccine group (*P* < 0.05) (Fig. 6F). Notably, T-bet expression on B cells, a marker of memory B cells (31), corresponded with CXCR3 expression, suggesting a mechanistic basis for enhanced antibody responses in the D_IP-10_ P_ALFQ_ vaccine group. Together, these data support the contention that T_h_1 skewing of CD4 T_fh_ cells may support higher anti-Env antibody levels.

To gain insights into the molecular mechanisms underlying successful antibody responses, we next determined the transcriptional signature in GC T_fh_ cells. To achieve this goal, we sorted naive CD4 cells, CD4 T_fh_ cells, and memory CD4 cells from the LNs of 3 D_IP-10_ P_ALFQ_ group animals with the highest gp140 serum IgG levels at week 8 after the 1st protein boost. These subsets were identified using the following markers: CD4^+^ CD95^−^ for naive cells, CD95^+^ CXCR5^+^ PD-1^+/++^ for Tfh cells, CD95^+^ CXCR5^+^ PD-1^−^ for memory Tfh cells, and CD95^+^ CXCR5^−^ PD-1^−^ for memory non-Tfh cells.

RNA samples meeting quality control checks were sequenced using a 3′-tag RNA-sequencing (RNA-Seq) library prep protocol at the UC Davis Genome Center using the Illumina HiSeq 4000 platform. Prior to the analysis of sequenced single-end reads, genes with fewer than 40 counts per million reads were filtered, leaving 7,086 genes. Differential expression analyses were conducted using the limma-voom Bioconductor pipeline (32) to compare the transcriptome profiles of antigen-experienced CD4 subsets to those of naive cells. Principal-component analysis of the 500 most variable genes based on coefficients of variation showed that CD4 transcriptomes clustered by cellular differentiation status, with memory CD4 T cells (both CXCR5^+^ and CXCR5^−^) sharing transcriptional signatures relative to naive and T_fh_ subsets (data not shown). To extract information on biologically relevant gene sets, we performed gene set enrichment analysis (GSEA) with the goal of determining biological pathways that were enriched in T_fh_ cells in the T_h_1 vaccine regimen. Genes regulating interleukin-12 (IL-12), tumor necrosis factor alpha (TNF-α), interferon gamma (IFN-γ), and IL-6 production were strongly enriched in T_fh_ cells. Consistent with the metabolic activity of effector cells and the functional capacity of T_fh_ cells, pathways regulating cellular metabolism, glucose homeostasis, and B cell proliferation were also enriched.

To determine the transcriptional activity of T_fh_ cells in the D_IP-10_ P_ALFQ_ vaccine group, we focused on differentially induced genes in T_fh_ cells relative to naive cells (*n* = 89; adjusted *P* value of <0.05) (Fig. 6G), of which the induction of key T_fh_ transcripts, including CXCR5, ICOS, and Bcl-6, was common to both T_fh_ cells and memory T_fh_ cells. A heat map shows the expression of genes differentially expressed in T_fh_ cells relative to naive cells across four sorted CD4 subsets. Consistent with the representation of D_IP-10_ P_ALFQ_ genes in the GSEA, T_fh_ cells showed higher expression levels of TBX21 and IFN-γ (Fig. 6G and H). The class IV semaphorin protein (SEMA4A), a costimulatory molecule expressed by D_IP-10_ P_ALFQ_ cells (33), was significantly induced, as was high-mobility-group box 1 (HMGB1), an inflammatory mediator regulating TNF and IL-6 production (34). The induction of IL-18 receptor (IL-18R) suggested the capacity of IL-18 to drive IFN-γ production within the GC (35). Likewise, we noted higher expression levels of receptor-interacting serine/threonine kinase 2 (RIPK2), which drives IFN-γ in T_h_1 cells and contributes to T_h_1 differentiation (36). The corresponding downregulation of IL-4R in T_fh_ cells indicated an enrichment of the T_h_1 program within T_fh_ cells in D_IP-10_ P_ALFQ_-vaccinated animals. This, together with the increased protein expression of CXCR3 within the GC, suggested that CD4 T cell help for humoral immunity was driven by T_h_1-T_fh_ cells in the D_IP-10_ P_ALFQ_ vaccine regimen.

### D_IP-10_ immunization induces systemic expansion of proinflammatory monocytes and enhances GC T_fh_ responses.

**FIG 7:**
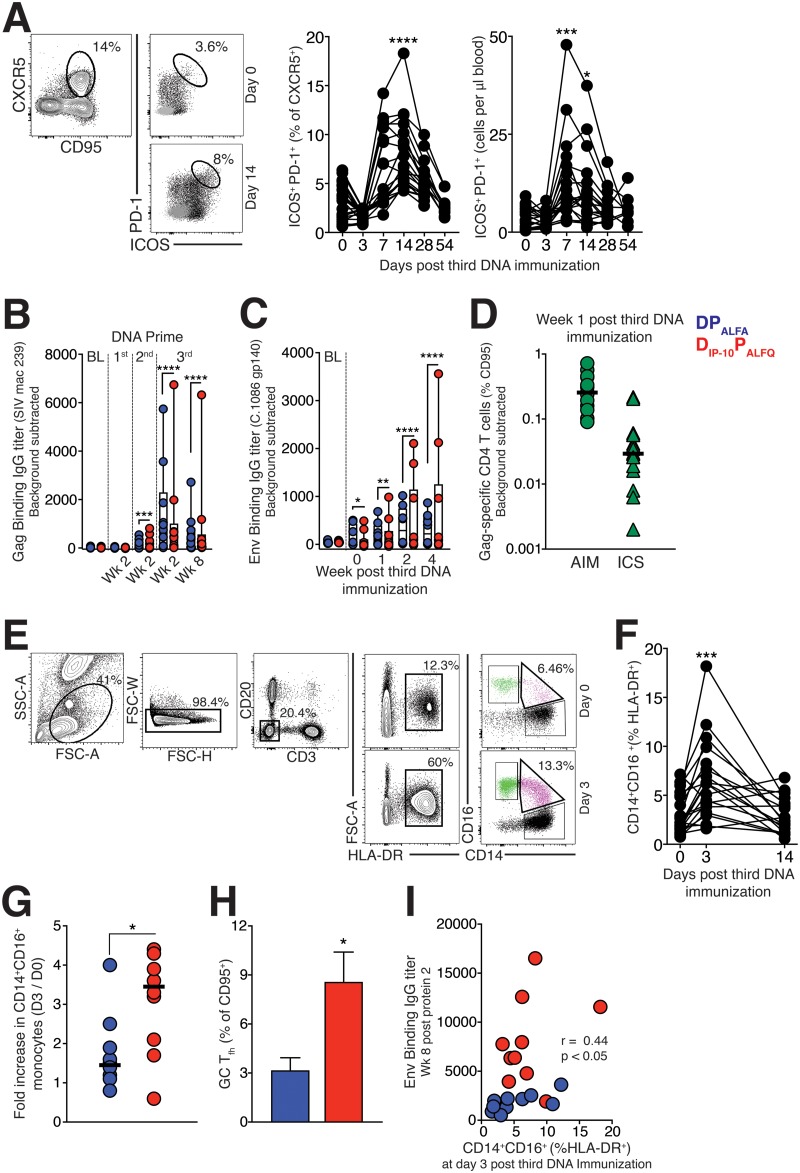
DNA_IP-10_ immunization induces systemic expansion of proinflammatory monocytes and enhances GC T_fh_ responses. (A) Gating strategy to identify activated CXCR5^+^ CD4 T cells in blood on day 0 and day 14 following the 3rd DNA immunization and transient accumulation of ICOS^+^ PD-1^+^ CXCR5^+^ cells in blood of all animals (*n* = 20) when expressed as relative frequencies (left) or absolute counts (right). (B and C) Kinetics of the SIVmac239 anti-Gag IgG response (B) and the C.1086 gp140-specific anti-Env IgG response (C) measured by an ELISA after DNA immunization at the indicated time points. Significance is indicated for all time points relative to baseline titers. (D) Gag-specific CD4 T cell responses measured at week 1 after DNA3 using AIM- and ICS (IFN-γ^+^ TNF-α^+^)-based assays. (E) Gating strategy to identify inflammatory CD14^+^ CD16^+^ monocytes in blood. (F) Frequency of CD14^+^ CD16^+^ (% HLA-DR^+^) monocytes following the 3rd DNA immunization. (G) Comparison of proinflammatory monocytes in blood of DNA- and DNA_IP-10_-primed animals. (H) Frequencies of GC T_fh_ cells in fine-needle aspirates of draining LNs from DNA- and DNA_IP-10_-primed animals on day 14 after the 3rd DNA immunization. (I) Spearman rank correlations between frequencies of proinflammatory monocytes in blood on day 3 and C.1086C gp140 IgG antibodies in serum on week 8 following the 2nd protein boost. Between-group differences were assessed using the Mann-Whitney U test (*, *P* < 0.05; **, *P* ≤ 0.01; ***, *P* ≤ 0.001; ****, *P* ≤ 0.0001).

Based on the increased frequencies of Env-specific T_fh_ cells and evidence for the induction of a T_h_1 transcriptome program in D_IP-10_ P_ALFQ_-vaccinated animals after the 1st protein boost, we sought to assess T_fh_ responses during the DNA priming phase. First, we evaluated blood to quantify activated CXCR5^+^ CD4 T cells in both vaccine groups (Fig. 7A). Based on the coexpression of ICOS and PD-1, activation markers induced upon T cell receptor (TCR) stimulation, the data showed that DNA immunization significantly increased the relative frequencies and absolute counts of ICOS^+^ PD-1^+^ CXCR5^+^ CD4 T cells in blood at day 14 (median frequencies of 3.38% at day 0 and 6.7% at day 14 [*P* < 0.0001] [*n* = 20]; absolute counts of 3.04 at day 0 and 8.7 at day 14 [*P* < 0.01] [*n* = 20]) (Fig. 7A) in both experimental groups, indicating that DNA delivery by electroporation was immunogenic.

Next, we assessed whether DNA immunization elicited humoral responses against SIV Gag and HIV Env proteins expressed in the plasmid. We found that detectable responses to Gag were observed in 45% of animals at week 2 of the 1st DNA prime, in 65% at week 2 following the 2nd DNA prime, and in all animals following the 3rd DNA prime (DNA3) (Fig. 7B [significance symbols compare immunization time points relative to the baseline]). Antibody responses to C.1086 Env were low and undetectable until the 2nd DNA prime (data not shown) but were observed in the majority of animals following DNA3 (Fig. 7C [significance symbols compare immunization time points relative to the baseline]). Gag and Env antibody titers were not significantly different between the vaccine regimens during the DNA primes. Based on the robust induction of anti-Gag antibody responses, we determined whether Gag-specific CD4 T cells were induced at week 1 following DNA3, when the CD4 effector response peaked. PBMCs were stimulated with pooled SIVmac239 Gag peptide pools and interrogated for the expression of activation markers (activation-induced marker [AIM] assay) and for the induction of cytokines (ICS). The AIM assay captured a higher proportion of Gag-specific CD4 T cells (Fig. 7D), and together, these data indicated that the DNA immunization was sufficiently immunogenic to prime T and B cell responses.

Based on the induction of antibody and T cell responses following DNA3, we next assessed whether a concomitant acute induction of proinflammatory monocytes (innate cells that drive T_fh_ responses) preceded the appearance of these cells in blood (37, 38). We quantified the frequencies of CD14^+^ CD16^+^ HLA-DR^+^ CD3^−^ CD20^−^ cells in blood (Fig. 7E and F) and discovered a rapid and robust expansion of proinflammatory monocytes in both vaccine groups, with significantly higher induction in the D_IP-10_ P_ALFQ_ vaccine group (Fig. 7G). Based on this, we asked if LN responses differed between vaccine groups. Strikingly, the GC T_fh_ cell frequencies within the fine-needle aspirates of the draining LN were higher in the D_IP-10_ P_ALFQ_ vaccine group following the 3rd DNA immunization (Fig. 7H). Notably, the greater inflammatory response was associated with increased levels of serum IgG antibodies, linking the innate immune response to the priming of effective CD4 T_fh_ help (Fig. 7I).

## DISCUSSION

The present study gives rise to three main conclusions: first that an HIV-1 vaccine platform designed to promote T_h_1-polarized T_fh_ cells increases the number of circulating Env-specific T_fh_ cells, enhances GC responses, increases anti-Env binding antibody titers in sera, and stimulates serum antibody effector functions; second that a T_h_1 vaccine regimen can elicit anti-Env vaginal and rectal IgA responses; and third that induction of high-avidity antibodies, reflective of productive GC responses, is engendered by a T_h_1 vaccine regimen. Collectively, the data suggest that adjuvant-induced stimulation of T_h_1-T_fh_ cell production during the vaccine prime and boost is an effective strategy to enhance the magnitude and functionality of the anti-Env antibody response.

Productive T cell responses critically depend on cytokine signals during priming, and recent studies demonstrate that monocyte-derived cytokines drive effective CD4 T cell differentiation and T_fh_ responses (38–40). Here, investigation of the kinetics of proinflammatory monocytes, cellular innate biomarkers of adjuvanticity, revealed a transient increase in CD14^+^ CD16^+^ monocytes in blood, with a higher relative increase in the D_IP-10_ P_ALFQ_ vaccine group. Strikingly, fine-needle aspirates of the draining LNs showed higher GC frequencies in the D_IP-10_ P_ALFQ_ vaccine group, indicating active/productive GC responses. Notably, the improved inflammatory response was associated with increased antibody magnitude, linking the innate immune response to the effective induction of CD4 T_fh_ cells. Although titers against Gag and Env were not significantly different between the vaccine regimens during the prime, it is possible that higher frequencies of memory B cells, which we did not quantify, were induced with the T_h_1 prime. Indeed, several recent studies show that potent priming of the immune response sets the stage for stronger boosting of cellular and humoral immunity in the settings of DNA prime and NYVAC boost and of adenovirus type 5 (Ad5) prime and NYVAC boost vaccine regimens (25, 41). The effectiveness of priming is not limited to CD4 T cells and B cells; a DNA vaccine targeting conserved elements of SIV Gag robustly primes cytotoxic T cells, which are effectively boosted following a long rest period (42, 43). These data open the possibility of a critical window of opportunity during the priming phase. This window can be exploited to prime for long-lasting, durable CD4 T cell, CD8 T cell, and antibody responses to HIV-1 vaccination.

HVTN studies 070 and 080 employed the IL-12 DNA-adjuvanted plasmid with the subtype B Pennvax-B (PV) DNA plasmid and showed 80% response rates after the third DNA vaccination in recipients of PV plus IL-12 (PV+IL-12), compared to a 44% response rate with the PV vaccine alone. A subsequent follow-up study demonstrated a robust recall of binding anti-Env antibody titers with ADCC activity following a modified vaccinia virus Ankara (MVA) boost in PV+IL-12 recipients (44, 45). Because IL-12 is a classic innate mediator of T_h_1 responses, the data suggest that an increase in T_h_1 GC T_fh_ cells may underlie the observed effects. Correspondingly, studies in rhesus macaques with an ALVAC prime and ALVAC+gp120 protein boost using SIV immunogens showed higher SIV Env titers with MF59 than with aluminum-adjuvanted protein boosts 2 weeks following the final immunization (46). While T_fh_ responses and memory antibody titers were not examined, a recent study in humans showed enhanced binding antibody titers 26 weeks after booster immunization with a T_h_1 glucopyranosyl lipid adjuvant formulated in a stable emulsion (GLA-SE)-adjuvanted malaria antigen relative to one formulated in aluminum (47). These studies, in conjunction with our report, provide support for the immune potential of T_h_1 T_fh_ cells in fostering high-magnitude antibody titers. In contrast, a study using a homologous subtype C protein immunization reported the induction of higher anti-Env antibody titers with aluminum hydroxide (Alhydrogel) than with AddaVax, an MF59 analog, in rabbits (48). Collectively, these data indicate the importance of detailed studies to understand the context in which T_h_1 responses are superior to mixed T_h_1 and -2 responses and how viral versus DNA vectors and subunit proteins influence this paradigm.

Our findings raise the question of the mechanisms underlying the D_IP-10_ P_ALFQ_ vaccine-mediated enhancement of T_fh_ responses. A few possibilities can be explored. IP-10 increases dendritic cell-T cell interactions, which could have favored T_fh_ differentiation (49). IP-10 also increases IL-6 production in B cells, which is known to support T_fh_ differentiation and enhance plasma cell survival (50). This, together with the potent immunostimulatory potential of the MPLA+QS-21 boost, may have synergized to enhance T_fh_ responses numerically and favored the T_h_1 differentiation program within T_fh_ cells (51). Indeed, GC T_fh_ cells induced following viral infections, where T_h_1 inflammatory responses predominate, express Bcl-6, Tbx21, IFN-γ, and IL-21, consistent with the induction of T_h_1-type T_fh_ cells (52). Transcriptomic analysis of T_fh_ cells following the 1st protein boost in the D_IP-10_ P_ALFQ_ vaccine regimen shows the coordinate expression of T_h_1-regulated genes, as evidenced by the enrichment of pathways related to IFN-γ signaling. It should be noted, however, that transcriptional analysis was performed on only 3 animals within the D_IP-10_ P_ALFQ_ group with the highest-magnitude antibody responses and therefore may have yielded false-positive targets and furthermore may not be representative of the GC T_fh_ signature elicited by the D_IP-10_ P_ALFQ_ vaccine regimen. Nonetheless, the higher relative expression levels of the T_h_1 chemokine receptor CXCR3 in GC T_fh_ cells and GC B cells and CXCR3 ligands in sera lend support to the gene expression data. Together, our transcriptomic and phenotypic data on T_fh_ cells indicate a role for adjuvant-induced quantitative (increased T_fh_ numbers) and qualitative (increased proportion of T_h_1 T_fh_ cells) effects on antibody magnitude. Mechanistic studies are needed to discern the respective contributions of increased T_fh_ numbers versus T_h_1 skewing of T_fh_ cells to antibody responses, as both of these characteristics are inextricably linked in the present study. Additionally, because our vaccine regimen differed by two components, IP-10 in the prime and ALFQ during the boost, further studies are needed to determine the specific role of the IP-10 prime versus the ALFQ boost in driving CD4 T_fh_ and antibody responses. This will enable us to address whether the T_h_1 boost synergized with the T_h_1 prime to enhance antibody titers and functionality or if a T_h_1 prime/T_h_1 boost alone would be sufficient to elicit the observed anti-Env antibody profiles.

While the D_IP-10_ P_ALFQ_ vaccine regimen increased the magnitude of anti-Env IgG titers in the vaginal mucosa, which correlated with decreased acquisition in each of the vaccine groups, our study was not powered to assess protection from acquisition across the vaccine regimens. Furthermore, the lack of an unvaccinated control group precludes the determination of vaccine efficacy and is a major caveat to the interpretation of acquisition outcomes. Therefore, more extensive larger-scale studies are needed to assess whether the D_IP-10_ P_ALFQ_ vaccine regimen induced protective antibodies with the capacity to mediate effective neutralization or antibody effector functions at the vaginal mucosa. Notably, in contrast to a previous study showing an increased risk of intrarectal acquisition with MF59 relative to an alum-adjuvanted protein immunization (46), the D_IP-10_ P_ALFQ_ vaccine regimen did not increase the risk of vaginal acquisition in the present study. While these studies differ in the routes of mucosal transmission, the difference in outcomes may also be attributed to the timing of exposure following the final immunization, i.e., 4 weeks in the previous study versus 20 weeks in the present study. It is possible that the presence of a higher frequency of CD4 T cell effectors at the rectal mucosa 4 weeks following immunization increased the acquisition risk, which could have contributed to the observed differences in outcomes. Indeed, higher frequencies of CCR5^+^ CD4 T cells in rectal mucosa were observed in vaccinated monkeys experiencing breakthrough infections than in those remaining uninfected following a low-dose intrarectal challenge (53). Therefore, whether increased immunogenicity detracts from protection is an important safety consideration in the use of T_h_1 adjuvants and other highly immunogenic vaccine platforms (46). This is particularly important as the HIV coreceptor CCR5 is primarily expressed on T_h_1 cells (12). Nevertheless, because T_h_1 cells also produce CCR5 ligands, it is important to determine the frequency of T_h_17 cells at the mucosal portals following immunization, as T_h_17 cells are preferential targets of infection within the vaginal mucosa (54). Another consideration is that the studies were performed on females and did not encompass the possible variability in vaccine responses between sexes. Therefore, going forward, it is critical to determine and confirm if a T_h_1 vaccine regimen will also enhance antibody responses in males.

In addition to adjuvant-dependent modulation of T_fh_ responses, our IgG subclass results also support the conclusion that the D_IP-10_ P_ALFQ_ and DP_ALFA_ vaccine regimens induced qualitatively different GC responses. This is also the first study to show that adjuvants can dramatically impact the IgG antibody subclass profile in rhesus macaques. We made the striking observation that while both vaccine regimens induced IgG1 antibodies to gp120, the DP_ALFA_ regimen generated much greater IgG4 responses. The T_h_2-promoting aluminum adjuvant is most likely responsible for the increased IgG4 in DP_ALFA_ animals because both vaccine groups received ALF liposomes. Rhesus IgG4 antibodies can mediate phagocytosis, but overall, they appear to have poor effector functions (55, 56), and the most functional IgG subclass in macaques has been reported to be IgG1 (56). Humans immunized with an alum-formulated HIV-1 gp120 protein have been found to develop IgG1 and IgG4 but not IgG2 and IgG3 antibodies (57). However, important functional differences in IgG subclass antibodies and FcγR biology between nonhuman primates and humans (58, 59), and the fact that rhesus IgG subclasses are numbered by serum abundance and not function, preclude direct comparisons between species. Another consideration is that differing antigen affinities between IgG subclasses for HIV-1 gp140 could confound quantitation, raising the possibility that subclass differences may be driven by differential affinities/epitope specificities rather than differential magnitudes. Therefore, more conclusive studies are needed to evaluate these possibilities.

Another notable observation was the induction, in D_IP-10_ P_ALFQ_-vaccinated animals, of a robust anti-Env vaginal IgA response with an accompanying decline in serum IgA antibodies after the 2nd protein immunization. This incongruity between vaginal and serum IgA responses was also observed in the DP_ALFA_ vaccine group, suggesting that ALF liposomes may have generated IgA plasmablasts that homed to the reproductive tract or possibly T_h_17-like T_fh_ cells, which promote IgA responses in mucosal LNs (60, 61). The T_h_1-biased ALF and ALFQ adjuvants have been reported to generate T_h_17 responses in mice, with ALFQ being more effective and additionally generating IgA antibodies (62). Future studies of T_fh_ cell subsets and IgA plasma cells in mucosal LNs will be required to determine if our T_h_1 vaccine regimen may have promoted IgA responses in the female reproductive tract, and in the rectum to a lesser extent, by generating T_h_17 cells.

In summary, our findings demonstrate that T_h_1-DNA priming substantially increases the frequency of Env-specific T_fh_ cells and that T_h_1-Env protein boosting results in greater production of anti-Env IgG1 antibodies with enhanced magnitude, breadth, avidity, and function. How this regimen can be further optimized to significantly enhance and induce robust tier 2 neutralizing antibodies is an important question that warrants further study.

## MATERIALS AND METHODS

### Rhesus macaques.

Twenty adult female colony-bred rhesus macaques (Macaca mulatta) were housed at the California National Primate Research Center and maintained in accordance with American Association for Accreditation of Laboratory Animal Care guidelines. All studies were approved by the University of California, Davis, Institutional Animal Care and Use Committee (IACUC). At study initiation, animals were 3.5 to 4.5 years of age with a median weight of 5.3 kg; were SIV negative (SIV^−^), simian T-cell leukemia virus negative (STLV^−^), and simian retrovirus negative (SRV^−^); had no history of dietary or pharmacological manipulation; and had intact ovaries.

### Immunizations and challenges.

DNA immunizations were administered via intradermal injection with electroporation utilizing the Ichor TriGrid array (Ichor Medical Systems) at weeks 0, 8, and 16. For each DNA immunization, two groups of 10 animals received 4 mg of the pGA2/JS2 plasmid DNA vector (63) encoding either SHIV C.1086 T/F Env plus interferon-induced protein 10 (IP-10) (group 1) or SHIV C.1086 T/F Env alone (group 2). Details of the SHIV DNA construct were described previously (64). At weeks 30 and 44, group 1 animals received boosts with 100 μg C.ZA 1197MB gp140 protein (Immune Technology) adjuvanted with 100 μg MPLA plus 50 μg QS-21 (ALFQ), and group 2 animals received 100 μg C.ZA 1197MB gp140 adjuvanted with 100 μg MPLA plus 600 μg aluminum (ALFA). The protein formulation (100 μg protein in a 500-μl formulation) was delivered in a 250-μl volume with 50 μg protein subcutaneously in each thigh during each of the two protein boosts. All animals were challenged at week 20 following the final protein immunization with a 1:4 dilution of SHIV.C.CH505 (stock at 189 ng/ml), obtained from George Shaw and Nancy Miller. The virus was diluted 1:4 in RPMI 1640 to obtain a challenge volume of 1 ml. Animals were positioned in a prone position, and a 1-ml syringe without a needle was used to inoculate the virus. Animals were challenged weekly, with 8 repeat doses, or until virus was detected in plasma.

### Adjuvants.

Dimyristoyl phosphatidylcholine (DMPC) and dimyristoyl phosphatidylglycerol (DMPG) saturated phospholipids, cholesterol (Chol), synthetic monophosphoryl lipid A (MPLA), and 3-deacyl monophosphoryl hexa-acyl lipid A (3D-PHAD) (Avanti Polar Lipids) were used as adjuvants. DMPC and Chol were dissolved in chloroform, and DMPG and MPLA were dissolved in chloroform-methanol (9:1). aluminum hydroxide (AH) (Alhydrogel) in a gel suspension was purchased from Brenntag. The QS-21 saponin was purchased from Desert King International and was dissolved in Sorensen phosphate-buffered saline (PBS) (pH 5.6).

Army liposome formulations (ALFs) containing DMPC, DMPG, Chol, and MPLA were prepared by the lipid deposition method. For vaccine preparations adjuvanted with ALFA, dissolved lipids were mixed in a molar ratio of 9:1:7.5:0.36 (DMPC to DMPG to Chol to MPLA) and dried by rotary evaporation followed by overnight desiccation. Liposomes were formed by using molecular-biology-grade water (Quality Biological), microfluidized, and sterile filtered, followed by lyophilization. One hundred micrograms of gp140 protein was adsorbed to 600 μg of Alhydrogel in PBS (pH 7.4) and incubated on a tilted roller at room temperature (RT) for 1 h prior to addition to lyophilized ALF. For vaccine preparations adjuvanted with ALFQ (ALF containing QS-21), lipids were mixed in a molar ratio of 9:1:12.2:0.36 (DMPC to DMPG to Chol to MPLA), dried, and rehydrated by adding Sorensen PBS (pH 6.2), followed by microfluidization and filtration. gp140 was mixed with ALFQ in a 1:1 volumetric ratio. Each vaccine dose in a 500-μl volume contained 100 μg MPLA (and 100 μg protein) and either 600 μg aluminum or 50 μg QS-21.

### Specimen collection and processing.

Lymph node (LN) biopsy specimens were obtained 2 weeks following each of the protein boosts and manually processed by disassociation through 100-μm cell strainers and washing in complete medium, as described previously (12). Two weeks after the 3rd DNA immunization, fine-needle aspirates of LNs were obtained using a 22-gauge needle, as previously described (65). PBMCs were isolated from whole blood collected in cell preparation tube (CPT) vacutainer tubes by density gradient centrifugation as previously described (12). For serum, coagulated blood was centrifuged at 800 × *g* for 10 min to pellet clotted cells, followed by extraction of fluid and storage at −80°C. Rectal and vaginal secretions were collected using premoistened Weck-Cel sponges and eluted as described previously (66).

### Serum IgG ELISA.

Serum IgG titers against HIV-1 C.1086 Env gp140 and Gag (SIVmac239) were determined by an ELISA. In brief, 96-well microtiter plates with a high binding capacity (Thermo Fisher) were coated overnight at 4°C with 1 μg/ml C.1086 Env gp140C from the NIH AIDS Reagent Program (ARP) or with SIVmac239 Gag (Immune Tech) diluted in 0.1 M carbonate-bicarbonate buffer (pH 9.2). Plates were washed with PBS containing 0.1% Tween 20 (PBST) and blocked with 5% (wt/vol) nonfat dry milk in PBS for 2 h at RT, followed by four washes with PBST. The standard (PG9 monoclonal antibody from the ARP) and serum samples were run at 3 dilutions/sample (1:50 to 1:450) in sample dilution buffer and incubated at RT for 2 h on a microplate shaker. After washing, the plate was incubated for 1 h with a 1:10,000 dilution of horseradish peroxidase (HRP)-conjugated goat anti-monkey IgG (Nordic MUbio). The plates were washed and then developed with 3,3′,5,5′-tetramethylbenzidine (TMB) substrate (Thermo Fisher), and the reaction was quenched with 2 N H_2_SO_4_ (Sigma). The absorbance was recorded at 450 nm with a reference filter at 570 nm using a Spectramax 5 plate reader (Molecular Devices). Baseline serum samples from each animal served as negative controls, and optical density (OD) values 2-fold above the baseline were considered positive and extrapolated to determine anti-Env antibody concentrations.

### Sodium thiocyanate avidity assay.

C.1086 Env gp140C-specific IgG antibody avidity was determined using a chaotropic displacement ELISA with sodium thiocyanate (NaSCN). Serum samples were incubated in duplicate at 6,000 pg per well for 2 h at RT. The plate was washed five times. For the dissociation step, one well of each sample was manually treated with 100 μl of 2 M NaSCN (Sigma-Aldrich) to dissociate antigen-antibody complexes, and a second well of the same sample was treated with PBS as a control. The plate was incubated for 15 min at RT, followed by washing three times. The plate was then developed as described above for the C.1086 gp140C ELISA. For each sample, antibody avidity was reported as an avidity index (AI) value (a percentage), which was calculated as the ratio of the absorbance in the well treated with NaSCN to that in the well treated with PBS.

### Biacore binding and avidity analysis.

Binding and avidity determinations were conducted using the Biacore 4000 surface plasmon resonance (SPR) system. The immobilizations were performed in a solution containing 10 mM HEPES and 150 mM NaCl (pH 7.4) using a standard amine-coupling kit, as previously described (23, 67). The CM5-S series chip surface was activated with a 1:1 mixture of 0.4 M 1-ethyl-3-(3-dimethylaminopropyl)carbodiimide hydrochloride (EDC) and 0.1 M *N*-hydroxysuccinimide (NHS) for 600 s (GE Healthcare). For the cyclic biotinylated V2 C.1086 peptide, 1 μM streptavidin (Life Technologies) in 10 mM sodium acetate (pH 4.5) (5,800 to 7,400 RU) was coupled for 720 s. The immobilized surface was then deactivated with 1.0 M ethanolamine-HCl (pH 8.5) for 600 s. Spot 3 in each flow cell was left unmodified to serve as a reference. Following surface deactivation, 0.06 to 1.5 μM cyclic biotinylated V2 C.1086 peptide was captured, resulting in two ranges of densities: high density (1,900 to 2,300 RU) and low/medium density (340 to 580 RU). For C.1086 gp140C, 0.56 to 15 μg/ml protein was immobilized directly on the CM5 sensor chip, resulting in four ranges of densities: very high density (9,800 to 10,100 RU), high density (3,400 to 4,100 RU), medium density (960 to 1,700 RU), and low density (240 to 670 RU). Following surface preparation, heat-inactivated serum samples were diluted 1:50 in running buffer (10 mM HEPES, 300 mM NaCl, and 0.005% Tween 20 [pH 7.4]). The diluted samples were injected onto the V2 peptide or gp140 protein surface for 320 s, followed by an 1,800-s dissociation period. The bound surface was then enhanced with a 240-s injection of 30 μg/ml of the secondary antibody goat anti-monkey IgG. To regenerate the bound surface, 175 mM HCl was injected for 70 s. For each serum sample or controls, 4 to 8 replicates were collected at a rate of 10 Hz, with an analysis temperature of 25°C. All sample injections were conducted at a flow rate of 10 μl/min. Data analysis was performed using Biacore 4000 Evaluation software 4.1 with double subtractions for an unmodified surface and buffer for the blank. Fitting was conducted using the dissociation mode integrated with Evaluation software 4.1.

### Binding antibody multiplex assay and sodium citrate avidity assay.

An HIV-specific serum IgG binding antibody multiplex assay (BAMA) was performed as previously described (68) with a panel of Env and V1V2 antigens: C.1086 gp140, CH505 T/F gp140, Con S (group M consensus) gp140, Con C (clade C consensus) gp140, and gp70-V1V2 clade B/Case A2 scaffolded protein. Samples were titrated in 5-fold serial dilutions starting at 1:80, and the binding magnitude is reported as the AUC. Positivity criteria (determined at a dilution of 1:80) were as follows: (i) a mean fluorescence intensity (MFI) of >100, (ii) an MFI higher than the Ag-specific cutoff (95th percentile of all baseline binding per antigen), and (iii) an MFI 3-fold higher than that of the matched baseline before and after blank/MLV subtraction. All BAMAs and avidity assays were performed in a blind fashion using magnetic beads. For avidity assays, samples were tested with and without sodium citrate (0.1 M, pH 3.0) at 2 dilutions for each antigen based on BAMA titration for maximum coverage of samples in the linear range of the assay. The dilutions were 1:80 and 1:400 for gp70-V1V2, 1:400 for C.1086 V1V2, 1:2,000 for CH505 T/F gp140, 1:2,000 for Con C gp140, and 1:10,000 for C.1086 gp140 and Con S gp140. Antibody avidity is reported as an avidity index, which was calculated as 100 × (MFI in the citrate-treated well/MFI in the untreated well). The avidity index is reported for sample-antigen combinations that were (i) identified as positive responders in the IgG BAMA and (ii) had an MFI within the linear range for the untreated sample.

### Neutralization.

Neutralization assays were performed as previously described (69) using TZM-bl cells. We measured neutralization activity against the tier 1 clade C pseudovirus MW965.26 using MLV-pseudotyped virus as an indicator of non-HIV-specific activity in the assay. Neutralization titers were measured at week 2 and week 8 after the 2nd protein boost and were considered to be positive for neutralizing antibody activity based on the criterion of a signal of ≥3× detected against the MLV negative-control virus. The majority of positive titers detected were against the tier 1 virus MW965.26, with occasional very weak neutralization titers against the tier 2 C.1086_B2 and SHIV CH505.375H viruses.

### Antibody-dependent cellular cytotoxicity.

The rhesus CD16^+^ human KHYG-1 NK cell line (effector cells) and CEM.NKR-CCR5-sLTR-Luc cell line (target cells) were provided by David Evans (University of Wisconsin) and were maintained in R10 culture medium consisting of RPMI 1640 supplemented with 10% fetal bovine serum, 25 mM HEPES, 2 mM l-glutamine, and 0.1 mg/ml Primocin (70, 71). The R10 medium for CD16^+^ KHYG-1 cells was additionally supplemented with cyclosporine (CsA) and interleukin-2 (IL-2) at concentrations of 1 μg/ml and 5 U/ml, respectively.

Luciferase-based ADCC assays were carried out as previously described, with some modifications (70). Two million CEM.NKR-CCR5-sLTR-Luc target cells were spinoculated with SHIV.C.CH505.375H.dCT (38 ng p27) for 2 h at 2,600 rpm at 30°C in the presence of 10 μg/ml Polybrene. Subsequently, the target cell-virus mixture was incubated overnight at 37°C with 5% CO_2_. The next day, virus was removed, and cells were incubated for another 72 h prior to the ADCC assay. For the ADCC assay, serum, effector cells, and target cells were plated in a 1:1:1 volumetric ratio. Serum was heat inactivated, diluted (1:50 dilution in R10 medium containing 10 U IL-2 per ml, with no CsA), and mixed with PBS-washed, infected target cells (1 × 10^4^ cells per well) and effector cells (5 × 10^4^ cells per well). Serum and cells were incubated overnight at 37°C in 5% CO_2_. Plates were then centrifuged at 1,800 rpm for 5 min at room temperature, and 100 μl of the supernatant was removed. The cell pellets were resuspended and mixed with 50 μl of the luciferase substrate reagent BriteLite Plus (PerkinElmer). Relative light units (RLUs) were recorded in black 96-well plates according to the manufacturer’s instructions, using a Synergy 2 microplate luminometer (BioTek). The percent ADCC activity of each tested serum sample (week 2 and week 8 after the 2nd protein immunization) was measured as the reduction in RLU compared to the animal’s week 0 preimmune serum (100% RLU). All samples were tested in triplicate, and experiments were performed twice.

### Antibody-dependent phagocytosis.

Serum antibodies were tested for their ability to enhance the phagocytosis of gp120-expressing beads by THP-1 cells using methods similar to those previously described (69). Briefly, 5 μl of 1-μm avidin-coated fluorospheres (Invitrogen) was labeled with 2 μg biotinylated anti-His tag antibody (Pierce) and then 3.5 μg His-tagged clade C gp120 Du151 protein (Immune Technologies) per plate. The gp120 beads and triplicate 5-fold dilutions of heat-inactivated serum in a 50-μl volume were then preincubated at 37°C in V-bottom plates. After 1 h, 2 × 10^4^ THP-1 cells in 50 μl were added to each well. After 5 h at 37°C in 5% CO_2_, the cells were washed in Ca^2+^- and Mg^2+^-free Dulbecco’s PBS (DPBS) and resuspended in 180 μl of warm 0.12% trypsin-EDTA. After 5 min at 37°C, the trypsin was removed, and the cells were resuspended in 1% paraformaldehyde. Fluorescence was evaluated using a FACSCanto instrument (BD Biosciences) and FlowJo software. Phagocytosis was measured by multiplying the percent fluorescent cells by their median fluorescence intensity. The phagocytic score was then calculated by dividing the phagocytosis of test samples by the average phagocytosis measured with preimmune serum.

### IgG subclass antibodies.

Ten rows of a 96-well Immulon 4 microtiter plate (VWR) were coated overnight at 4°C with 50 ng per well of C.1086 gp120 Δ7 K160N protein (72) in PBS. The remaining 2 rows were coated with duplicate 2-fold serial dilutions of rhesus IgG1, IgG2, IgG3, or IgG4 (Nonhuman Primate Reagent Program) starting at 25 ng/ml in PBS to generate a standard curve. Plates were washed with PBS containing 0.05% Tween 20 and blocked for 30 min at RT with reagent buffer (0.1% bovine serum albumin in wash buffer). Twofold or 3-fold dilutions of serum in reagent buffer were then added to the wells coated with gp120. Reagent buffer was added to wells coated with the standard. Following overnight storage at 4°C, the plate was washed and reacted for 1 h at 37°C with 1 μg/ml of the relevant monoclonal antibody from the Nonhuman Primate Reagent Resource: anti-rhesus IgG1 (mouse IgG2a clone 3C10.3), anti-rhesus IgG2 (mouse IgG1 clone 3C10), anti-rhesus IgG3 (mouse IgG1 clone 2G11), or anti-rhesus IgG4 (mouse IgG1 clone 7A8). These antibodies were raised to react specifically with the respective rhesus IgG subclasses and show negligible reactivity with other subclasses, and the specificity of 7A8 was further confirmed in our laboratory. The plate was then consecutively washed and treated with 100 ng/ml of biotinylated goat anti-mouse IgG1 or IgG2a for 1 h at 37°C, Neutralite-avidin peroxidase for 30 min at RT, and TMB (all from SouthernBiotech). The absorbance was recorded at 370 nm. SoftMax Pro software (Molecular Devices) was used to construct a standard curve and determine concentrations of antibody. Preimmune serum samples had <10 ng/ml of antibody in these assays.

### Mucosal antibodies and serum IgA.

A BAMA with C.1086 gp140 K160N-labeled magnetic beads (MagPlex; Bio-Rad) was used as previously described (72) to measure concentrations of antigen-specific IgG in secretions and IgA in both secretions and serum depleted of IgG. Briefly, beads reacted with dilutions of the standard (73) and specimens at 1,100 rpm at 4°C overnight were washed and developed with biotinylated anti-monkey IgG or -monkey IgA (Rockland) followed by phycoerythrin-labeled Neutralite-avidin (SouthernBiotech). The construction of standard curves and interpolation of antibody concentrations were done using Bioplex Manager software after measurement of fluorescence with a Bioplex 200 instrument (Bio-Rad). Concentrations of gp120-specific IgG or IgA in secretions were divided by the total IgG or IgA measured in the sample by an ELISA (74) to obtain the specific activity (nanograms of IgG or IgA antibody per microgram of total IgG or IgA).

### Activation-induced marker assay.

Cells were stimulated with overlapping peptide pools of HIV consensus C and HIV-1 C.1086 Env gp140C proteins and SIVmac239 Gag in activation-induced marker (AIM) medium as previously described (30). All antigens were used at a final concentration of 2 μg/ml in a stimulation cocktail made using 0.2 μg of CD28 and 0.2 μg of CD49d costimulatory antibodies per test. Unstimulated controls were treated with volume-controlled dimethyl sulfoxide (DMSO) (Sigma-Aldrich). Tubes were incubated in 5% CO_2_ at 37°C overnight. Following an 18-h stimulation, the cells were stained, fixed, and acquired the same day. A phenotype panel on LNs and PBMCs was performed using standard flow cytometry assays (12).

### Serum cytokines.

A Legendplex assay (BioLegend) was performed to evaluate cytokines in rhesus macaque sera. The assay was performed according to the manufacturer’s protocol. Samples were acquired on a BD LSR Fortessa cell analyzer.

### Flow cytometry and cell sorting.

Cell staining and sorting were performed as previously described (12). Fluorescence was measured using a BD FACSymphony cell analyzer with FACSDiva version 8.0.1 software. Compensation, gating, and analysis were performed using FlowJo (versions 9 and 10). Cell sorting was performed using a BD FACSAria III cell sorter. Reagents used for flow cytometry are listed in Table 1.

**TABLE 1:** Antibodies and viability dye used for cell staining^*a*^

Reagent	Fluorochrome	Clone	Catalog no.	Source
CD28	Unconjugated	CD28.2	555725	BD Biosciences
CD49d	Unconjugated	9F10	555501	BD Biosciences
CD3	AF700	SP34-2	557917	BD Biosciences
CD4	BV650	L200	563737	BD Biosciences
CD8	BV510	SK1	563919	BD Biosciences
CD20	Pacific blue	2H7	302328	BioLegend
CXCR5	PE	MU5UBEE	12-9185	Thermo Fisher
CD25	APC	BC96	20-0259	Tonbo Bio
CD134	BV786	L106	744746	BD Biosciences
CD95	PE-Cy5	DX2	564710	BD Biosciences
Live/dead	Aqua/BV510	N/A	L-34966	Thermo Fisher
ICOS	BV786	C398.4A	313534	BioLegend
CXCR3	APC	IC6	550967	BD Biosciences
PD-1	APC-Cy7	EH12.2H9	329922	BioLegend
IP-10	PE	J034D6	519503	BioLegend
Bcl-6	AF488	K112-91	561524	BD Biosciences
Ki-67	BUV 395	B56	564071	BD Biosciences

a PE, phycoerythrin; AF, Alexa Fluor; BV, Brilliant violet; BUV, Brilliant ultraviolet; Cy, cyanine; APC, allophycocyanin; N/A, not applicable.

### RNA sequencing and bioinformatics.

RNA was extracted from sorted subsets, and DNA-free RNA was quantified and assessed for quality prior to sequencing. RNA samples with visible peaks, a 260/280 absorbance ratio of between 1.8 and 2.1, and an RNA integrity number of >7 were sequenced using 3′ Tag-Seq gene expression profiling on the Illumina HiSeq sequencer at the DNA Technologies and Expression Analysis Core Laboratory at the UC Davis Genome Center. Samples were barcoded and run in a single HiSeq lane. The quality of data was verified using the Illumina SAV viewer; this included verifying low error rates based on alignments of the standard Illumina PhiX spike and removal of PCR duplicates after alignments. Adapter trimming, quality control (QC) of sequencing data, and demultiplexing were performed by the UC Davis Bioinformatics Core. After read filtering, reads were mapped to a reference genome using HISAT-aligner. On average, 82.17% of reads were mapped (∼55 to 61% uniquely mapped), and the uniformity of the mapping result for each sample indicated comparability between samples. Prior to differential gene expression analysis, genes with fewer than 40 counts per million reads were filtered, leaving 7,086 genes. Differential expression analyses were conducted using the limma-voom Bioconductor pipeline (32).

### Statistical analysis.

Statistical analysis was performed using GraphPad Prism 7. Results between groups were compared using the two-tailed nonparametric Mann-Whitney rank sum test. Within-group comparisons, such as antibody levels at different time points, were done using the two-tailed Wilcoxon matched-pairs signed-rank test. For correlation analysis, the two-tailed Spearman rank correlation test was used.

### Data availability.

The data discussed in this publication have been deposited in NCBI's Gene Expression Omnibus and are accessible through GEO Series accession number GSE141707.
